# Retraction: Qian, W., et al. In Vitro Antibacterial Activity and Mechanism of Vanillic Acid against Carbapenem-Resistant *Enterobacter cloacae*. *Antibiotics* 2019, *8*, 220

**DOI:** 10.3390/antibiotics9060322

**Published:** 2020-06-12

**Authors:** Antibiotics Editorial Office

**Affiliations:** MDPI, St. Alban-Anlage 66, 4052 Basel, Switzerland; antibiotics@mdpi.com

The authors have been made aware that two figures were published in two papers: Figure 5A in paper [1] is duplicated as Figure 3D in published paper [2], and Figure 3A in paper [1] is duplicated as Figure 3A in published paper [2]. The authors state this was due to carelessness. In order to preserve academic integrity, the *Antibiotics* Editorial Office has taken the decision to retract the paper with the consent of the authors. The authors assert that the findings, that vanillic acid exerted a significant antibacterial activity against carbapenem-resistant *Enterobacter cloacae* (CREC), are nevertheless reliable [1].

The Editor-in-Chief has checked the case and communicated with the authors. The decision to retract has been taken in agreement with the authors of the paper [1]. The *Antibiotics* Editorial Office and authors apologize to the readers of *Antibiotics* for any inconvenience caused.

To ensure the addition of only high-quality scientific works to the field of scholarly publication, this paper [1] is retracted and shall be marked accordingly. MDPI is a member of the Committee on Publication Ethics (COPE) and takes very seriously the responsibility to enforce strict ethical policies and standards.
